# Special Issue Introduction: Role of Epigenetic Gene Regulation in Brain Function

**DOI:** 10.3390/genes8070181

**Published:** 2017-07-13

**Authors:** Dennis R. Grayson

**Affiliations:** Center for Alcohol Research in Epigenetics, Department of Psychiatry, College of Medicine, University of Illinois at Chicago, Chicago, IL 60612, USA; Dgrayson@psych.uic.edu

In 1957, Conrad H. Waddington published a paper in which he demonstrated the inheritance of an acquired characteristic in a population in response to an environmental stimulus [1]. In so doing, he exploited the concept of plasticity in the population to discover what characteristics might be influenced by interactions with the environment. As the founding father in the field, Waddington’s ideas laid the foundation for the field of epigenetics, a term he had coined. Nowadays, we understand that these changes occur through mechanisms that involve either DNA methylation or histone modifications that turn genes on and off, such as histone acetylation and methylation. Epigenetics is the study of potentially heritable changes in gene expression that do not involve changes to the underlying DNA sequence. This definition is attributed to Waddington [2] as he was trying to combine genetics and developmental biology. In 2008, scientists meeting at Cold Spring Harbor devised a consensus concept of epigenetics that was: the stably heritable phenotype resulting from changes in a chromosome without alterations in the DNA sequence [3]”. While this definition holds true to the original meaning of Conrad Waddington, common day use of the term often refers simply to heritable changes in chromatin remodeling.

While there were multiple studies on the epigenetics of various brain cancers early on, some of the more poignant papers on epigenetics and the brain were initiated with studies of maternal behaviors. In 2004, Michael Meaney and co-workers reported that increased pup licking and grooming, and arched-back nursing by rat mothers alters the epigenome at the glucocorticoid receptor gene promoter in the hippocampus of their offspring [4]. That is, the glucocorticoid receptor gene promoter in the hippocampus of offspring of high licking grooming and arched-back nursing was less methylated than the promoter in low licking and grooming and arched back nursing mothers [4]. These results emerged over the first few weeks of life and persisted until adulthood [4]. Moreover, the glucocorticoid receptor promoter methylation was reversed by cross-fostering. They further showed that the DNA methylation changes were reversed by the administration of a histone deacetylase (HDAC) inhibitor and this reversal involved a region of glucocorticoid receptor that was linked with the binding of the transcription factor, Nerve growth factor 1A (NGF1A) [4]. Since the initial findings were published, numerous reports have investigated glucocorticoid receptor methylation in relation to early life stress [5]. We now know that there are many components that contribute to the quality of the early life environment including parents, siblings and other caregivers. Absence of family members, as well as the lack of food, sensory stimulation, social support, etc., may have a sustained impact on divergent developmental trajectories [6].

A role for DNA methylation in regulating transcription was proposed some 40 years ago [7,8]. DNA methylation was initially hypothesized to play an important neuron-specific function aside from its role in replication when it was identified in non-mitotic neurons of the brain [9]. We now know that following depolarization, changes in neuronal gene expression are accompanied by changes in DNA methylation and other epigenetic marks that alter the chromatin landscape. We also now know that in addition to 5-methylcytosine, additional cytosine modifications, including 5-hydroxymethylcytosine, 5-formylcytosine and 5-carboxylcytosine, exist and are stable intermediates in the DNA demethylation process. DNA methylation is catalyzed by a family of DNA methyltransferases [10], while the additional oxidation states are formed through the action of the TET family of methylcytosine dioxygenases [11]. In general, DNA methylation often correlates with transcriptional repression, while hydroxymethylation correlates more with transcriptional activation [12]. However, genome-wide methyl mapping studies suggest that the function of DNA marks tend to vary with genomic context and location of the mark [12,13].

A role for epigenetics in the etiopathopathophysiology of schizophrenia and other mental disorders was proposed, in part, to account for the complex heritability observed in various psychiatric pedigrees. The epigenetics of schizophrenia was first proposed in a book by Gottesman et al, titled *Schizophrenia: The Epigenetic Puzzle* [14]. In a paper of the same title, Petronis argues that facets of epigenetics need to be evaluated in the context of the four theories regarding the origins of schizophrenia: neurodevelopmental, dopamine dysfunction, viral, and genetic anticipation with unstable DNA [15]. Each of these theories regarding the origins of Sz are influenced by epigenetic mechanisms introduced by interactions with the environment. The authors note that many studies have investigated genetics and epigenetics separately despite current thinking that suggests interactions between genes and a deleterious environment are necessary but not sufficient for the disease. This bias in approach still persists. In a subsequent paper, they argue that DNA acts much like the hardware of a computer and epigenetics is akin to software which makes it run [16]. We now have come to appreciate that exposure to a large number of environmental factors, particularly during pregnancy and early post-natal life, often results in the acquisition of epigenetic marks that can be important in the pathogenesis of Sz. These factors include birth and obstetric complications, history of infection, place and season of birth, physical and psychological abuse and cannabis use during adolescence [17,18,19]. The findings suggest that preclinical studies need to focus on how specific environmental factors impact gene expression programs during brain development. These kinds of approaches are now underway in various labs investigating schizophrenia [18] and also autism spectrum disorder in the context of environment factors [20,21].

Interest in the efficacy of epigenetic drugs in the treatment of psychiatric disorders has been fueled by the finding that valproic acid (VPA, sodium valproate or divalproex sodium) acts to inhibit HDACs [22,23]. VPA was serendipitously discovered as a drug that would prevent pentylenetetrazole-induced seizures in rats [24] and was subsequently marketed as an anticonvulsant drug. Reports of the use of VPA in the treatment of bipolar illness first appeared in the literature in the late 1970s and early 1980s. It was shown to be effective in the treatment of acute mania and is often used in the maintenance treatment of bipolar disorder [25]. We will expand upon this in the following section. A role for HDACs in the pathophysiology of SZ was hypothesized several years ago. There have been genetic studies showing associations of the Class 1 HDACs 2 and 3, the Class II HDACs 9 and 10, and the Class IV HDAC 11 with SZ in certain populations [26,27,28,29]. An analysis of microarray data obtained from post-mortem SZ subjects showed that there was increased expression of HDAC1 in the prefrontal cortex [30]. In addition, analysis of gene expression patterns in the hippocampus and medial temporal cortex of an independent group of SZ post-mortem subjects show increased HDAC1 mRNA levels [31]. In contrast, analysis of the dorsal lateral PFC of a large number of post-mortem SZ subjects demonstrated that the expression of HDAC2 but not HDAC1 mRNA is reduced [32]. More recently, evidence supports a role for the inhibition of HDAC2 as a new therapeutic target for SZ patients, particularly those patients that have been treated with atypical antipsychotics for long periods of time [33,34]. Other HDACs, such as HDAC9 [28] have also been implicated in the context of SZ.

With this Special Issue of the journal *Genes*, we aim at presenting recent research and developments in the field. Special attention has been given to articles on genes and how they change in response to epigenetic effects of drugs of abuse, aging, psychiatric disorders, learning, maternal factors and adverse environments and normal neuronal and brain function. While these represent a diverse set of studies, the underlying theme is that environmental factors impact the brain through changes in DNA methylation which alter gene expression and cellular/neuronal function. In the near future, preclinical epigenetics will need to focus on specific environmental factors and a better understanding of transcriptomic changes occurring in multiple brain regions as a consequence of exposure.
